# Granulomatous Mastitis With Breast Abscess Caused by Salmonella

**DOI:** 10.7759/cureus.39585

**Published:** 2023-05-28

**Authors:** Lina Alsaket, Sara Hassan, Nahla Eltai, Emad Elmagboul, Amal Alobadli

**Affiliations:** 1 Clinical Imaging, Hamad Medical Corporation, Doha, QAT; 2 Surgery, Hamad Medical Corporation, Doha, QAT; 3 Microbiology, Qatar University, Doha, QAT; 4 Pathology and Laboratory Medicine, Hamad Medical Corporation, Doha, QAT

**Keywords:** salmonella, hyperprolactinemia, granulomatous mastitis, empty sella syndrome, breast abscess

## Abstract

Granulomatous mastitis (GM) is a rare disease that occurs in young premenopausal women, is mostly idiopathic, and is less likely to be caused by infection and trauma. It is also strongly associated with pregnancy, lactation, and hyperprolactinemia. GM superimposed by infection with abscess formation caused by Salmonella is extremely rare. Upon reviewing the literature, our case is considered to be the first reported case globally. Most breast abscesses are caused by Staphylococcus aureus.

## Introduction

Granulomatous mastitis (GM) was first described by Kessler and Wolloch in 1972 [1]. It can be caused by a wide range of etiologies and is associated with different predisposing factors; one of them, hyperprolactinemia, can occur due to different etiologies, as an example of Empty Sella syndrome as in our case presentation.

Clinically, patients with GM can present with pain, redness, swelling, breast mass, or skin ulceration. Differentiation from breast cancer can sometimes be difficult and requires tissue diagnosis to rule out malignancy.

Radiologically, mammograms or ultrasounds can reveal masses, asymmetries, skin thickening, or fluid collection. Pathologically, it is a chronic noncaseating granulomatous inflammation of the breast composed of giant cells, leukocytes, epithelioid cells, macrophages, and microabscesses [2]. Most GM can be caused by an unknown etiology; however, it is rarely caused by trauma, immunosuppressants, tuberculosis, foreign bodies, or infections. Infections include parasitic, bacterial, and viral infections.

A wide review of the literature suggests that GM can be predisposed to hyperprolactinemia, as the breast ducts and lobules are filled with thick content, which causes obstruction associated with static secretions that may either become infected or escape into the perilobular stroma, leading to a T-cell-mediated immune response and the formation of granulomas. Indeed, several infectious agents have been identified in patients with GM, most notably Corynebacterium spp. [1].

Breast abscess formation is an uncommon finding in GM and is rarely caused by infectious agents, such as Salmonella. As gram-negative bacilli, Salmonella species are mainly associated with extraintestinal or intestinal diseases which present with gastrointestinal symptoms, mainly abdominal pain, diarrhea, nausea, and vomiting, in addition to fever; however, it can be complicated by abscesses in different organs, but very rarely in the breast. We discuss this case to identify the importance of fluid culture in different breast abscesses to rule out a rare cause and aid with treatment options.

## Case presentation

A 30-year-old female was diagnosed with hyperprolactinemia (Empty Sella syndrome) caused by cabergoline many years prior. The patient presented to the primary care center in Qatar with redness, swelling, and pain in the right breast with no fever for one week, and was diagnosed with cellulitis. She received Augmentin (Amoxicillin/Clavulanic acid) (500 mg for seven days) and showed only minimal improvement. A COVID-19 test was performed during the illness and was negative. She continued to experience redness, swelling, and pain, with slight localized swelling on the lateral side of the areola of the right breast and some oozing. She denied fever or chills, labs were performed and showed elevated white blood cells (11.0 x10^3^/µL) and CRP (30.0 mg/L). She started another course of Augmentin (Amoxicillin/Clavulanic acid) but developed a skin rash as a result of an allergy to Augmentin (Amoxicillin/Clavulanic acid). She was switched to clindamycin (oral 450 mg TID for seven days), but her main symptoms increased, and she presented two days later to the emergency with worse complaints. Clinical examination performed in the ED showed a large area of redness involving the whole outer quadrant of the right breast and the areola with an open sinus with blood-stained pus. Clinical aspiration and fluid culture showed no growth, and a breast ultrasound was ordered.

**Figure 1:**
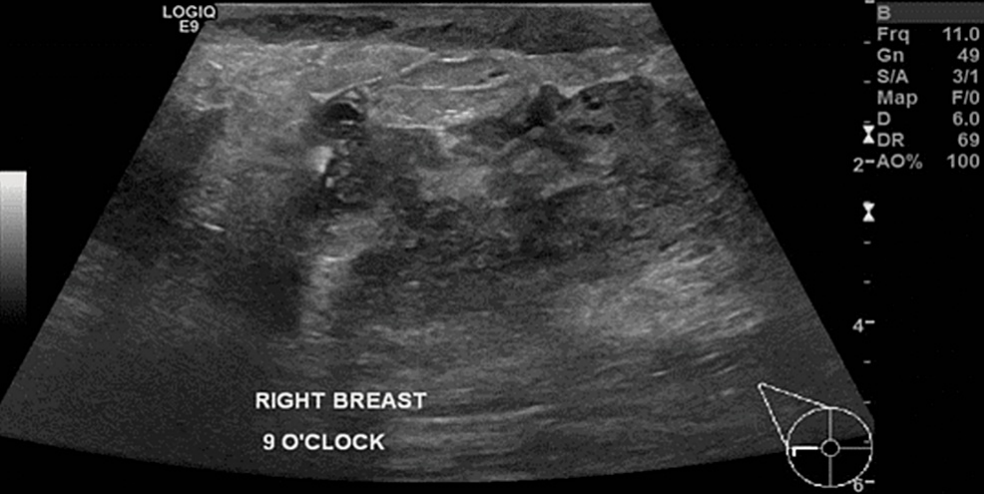
Right breast ultrasound showed heterogeneity with fluid collections more prominent at outer quadrant of right breast.

**Figure 2:**
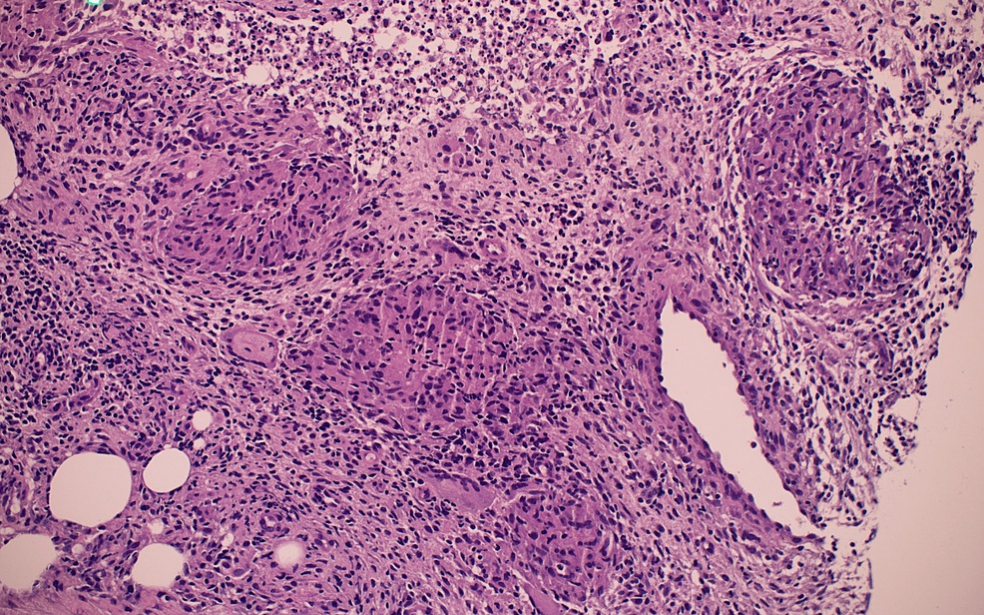
Histopathology slide showed low magnification (x20) which showed chronic inflammation with multiple non-necrotizing granulomas. Special stains for acid-fast bacilli and fungi are negative.

**Figure 3:**
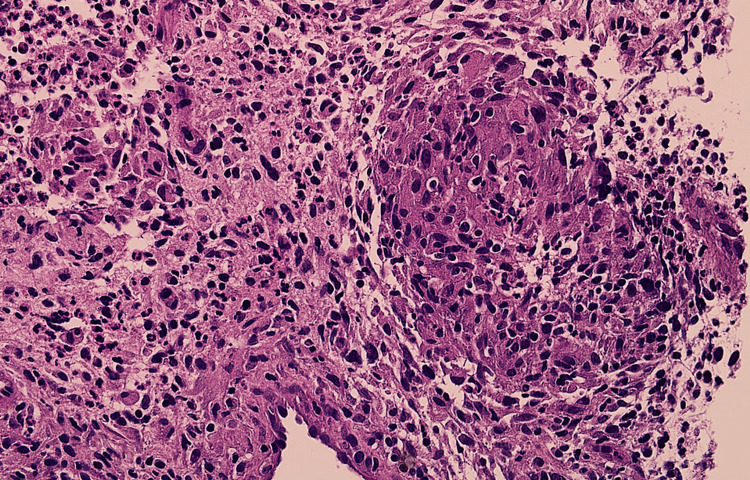
Histopathology slide showed high magnification (x40) which showed chronic inflammation with multiple non-necrotizing granulomas. Special stains for acid-fast bacilli and fungi are negative.

Right breast ultrasound was performed and showed multiloculate fluid collections in the outer right breast (Figure 1). Aspiration followed by biopsy under ultrasound guidance and histopathology showed GM (Figures 2, 3) with Corynebacterium kroppenstedtii growth culture. Staining for acid-fast bacilli (AFB) and fungi (Grocott) was negative, and results were negative for carcinoma in situ, and invasive carcinoma.

**Figure 4:**
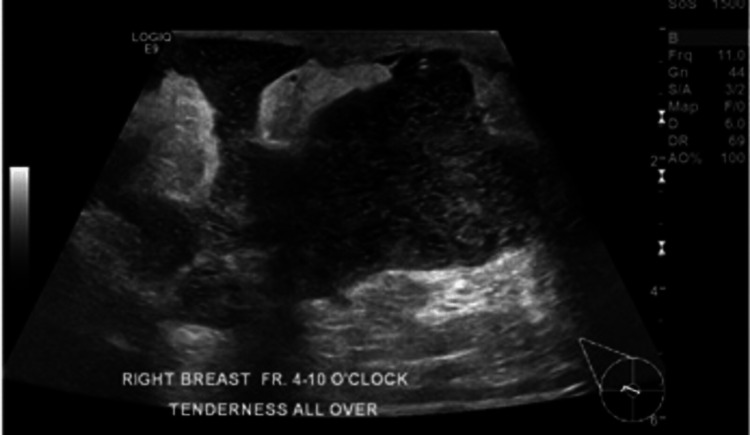
Right breast ultrasound showed new fluid collections at the retroareolar area of the right breast from 10:00 to 4:00.

**Figure 5:**
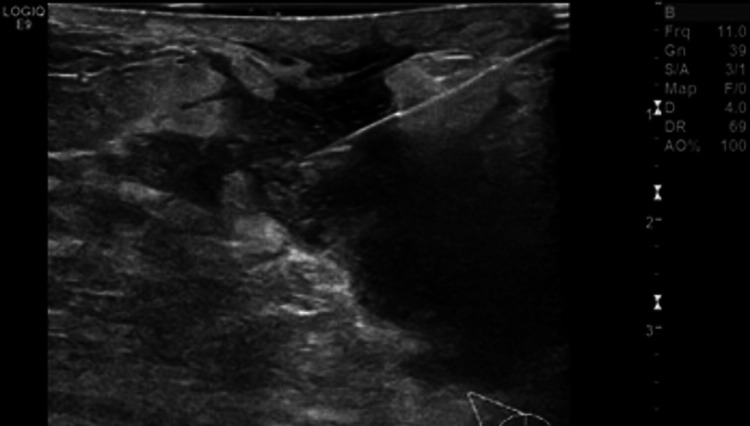
Ultrasound-guided aspiration of the fluid collection using 18G needle.

She was started on prednisolone 50 mg for two weeks then tapered to 25 mg for another two weeks. She had an initial response, as the sinuses in the breast diminished, but only a lump sensation persisted. When the prednisolone dose was tapered off, a new lump appeared near the right nipple. Another breast ultrasound was performed and showed new fluid collections at the retroareolar area of the right breast (Figure 4). Aspiration was again performed under ultrasound guidance (Figure 5), and culture showed Salmonella enteritidis. The aspirated breast fluid that was received in the microbiology laboratory at HMC was examined microscopically and profuse polymorph leukocytes without epithelial cells, but scanty gram-negative bacilli were detected. After overnight incubation of the fluid directly and from enriched cultures on MacConkey agar at 37ºC, non-lactose fermenter colonies were identified, which tested negative for oxidase and produced hydrogen sulfide gas on triple sugar agar.

 

Salmonella species were identified using automated machines, including Bruker Biotyper Matrix-Associated Laser Desorption Ionization-Time of Flight Mass Spectrometry (MALDI-TOF MS), and confirmed as Salmonella species by Phoenix TM. Serologically, it was identified as Salmonella group D1(O:a, H:1,2, Vi) using a panel of agglutinating sera from Remel™ Agglutinating Sera, Salmonella O/Vi, and BD Difco™ Salmonella O Antisera.

Further, molecular identification of Salmonella as S. enteritidis ST 11 was performed by sequencing the Salmonella seven housekeeping genes (aroC, thrA, purE, his D, hemD, sucA, dnaN) and analyzed by multilocus sequence typing, MLST (https://pubmlst.org/organisms), and NCBI blast (https://blast.ncbi.nlm.nih.gov/).

Antibiotic susceptibility testing was performed using Phoenix TM and minimum inhibitory concentrations were interpreted according to CLSI guidelines. Stool and urine cultures were taken, and analysis showed that they were negative for Salmonella.

Fast tapering of prednisolone was started, and a consultation was sent to the Centre for Disease Control and Prevention in Hamad Medical Corporation, and Trimetho/Sulfa (Oral 160 mg/800 mg q12hr for 14 days) was started. At one-month clinical follow-up, the lump on the right breast appeared very small and could be felt on/off with no pain or skin redness. All the sinuses healed without pus discharge. Ultrasound was performed and showed a small residual abscess collection. Ultrasound-guided aspiration was performed, and the culture showed no growth.

**Figure 6:**
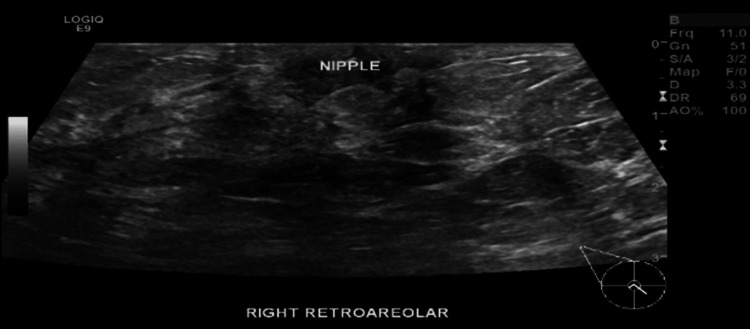
Right breast ultrasound showed complete resolving of the breast abscess

A trial of two doses of intralesional methylprednisolone 120 mg (40 mg/mL) was injected into the right breast, and a complete response on ultrasound was noted after three months (Figure 6). The patient is now doing well, and is fully recovered with no complications.

## Discussion

GM is an inflammatory disease that can be triggered by hyperprolactinemia, caused by pituitary adenoma, Empty Sella Syndrome, and antidepressant therapy, and is considered to be an immune suppression disease in the breast that requires conservative treatment. In most cases, steroids are administered (different routes of injection including IV, oral, or intralesional). GM can be superimposed in very unusual cases of breast abscess caused by different types of infection, mostly bacteria, such as Staphylococcus aureus [3], but is extremely rare with Salmonella, as in our case.

The presumptive causes of GM, including alpha-1-antitrypsin deficiency, oral contraceptives usage, pregnancy, lactation, and hyperprolactinemia have been reported Interestingly, hyperprolactinemia whether primary or secondary, might play an important role in GM. IGM in non-pregnant women associated with high serum prolactin levels can have been rarely reported associated with pituitary adenoma, phenothiazine-induced hyperprolactinemia, and metoclopramide-related galactorrhea with blunt trauma [4].

A close association between IGM and hyperprolactinemia has been reported [5]. Also, according to multiple reports, prolactin levels are important in recurrent cases [5]. Salmonellae are gram-negative bacilli that belong to the Enterobacteriaceae family. Disease caused by Salmonella organisms can be divided into two categories: typhoidal and nontyphoidal. The reservoir for typhoidal disease is humans, but nontyphoidal salmonellae are widely distributed among animals. In humans, nontyphoidal Salmonella infections are most often associated with food products; the remainder are nosocomial infections or are acquired from pets [6].

The reported focal manifestations include pyelonephritis, empyema, endocarditis, meningitis, brain abscess, thyroiditis, osteomyelitis, cutaneous abscesses, and pyomyosis. More than 50% of such patients have bacteremia. Localized disease is thought to result from both overt and silent bacteremia with subsequent seeding of distant sites, often areas of preexisting disease [7,8].

Salmonella species pathogenesis is considered a generalized or localized disease and can include various organs, such as the gastrointestinal tract, reticuloendothelial system, brain, thyroid, breast, and skin [8], and presents with a wide range of symptoms. It mainly occurs in epidemic countries and is unusual in developing countries such as Qatar. Treatment options are localized, either by medical or surgical routes.

Our case is unique in that the patient had a personal history of hyperprolactinemia, developed GM in the breast, and was superimposed by Salmonella breast abscesses. This highlights the importance of continued follow-up by imaging and additional culture when new symptoms appear.

## Conclusions

GM is a complicated disease that requires special attention and the use of different types of treatment. Our case of Salmonella spp. causing breast abscesses with a background of GM disease was considered the first report in the literature. This report highlights the importance of continuous follow-up imaging and aspiration of abscesses with new cultures, particularly when new symptoms arise.
